# Pharmacological Inhibition of cFLIP Targets Breast Cancer Stem Cells

**DOI:** 10.29245/2578-2967/2025/2.1210

**Published:** 2025-08-08

**Authors:** Rhiannon French, Olivia Hayward, Andreia Ribeiro da Silva, Timothy Robinson, Gillian Seaton, Richard Clarkson

**Affiliations:** 1 European Cancer Stem Cell Research Institute, Cardiff University School of Biosciences, Hadyn Ellis Building, Maindy Road, Cardiff CF24 4HQ; 2 Bristol Medical School (PHS), University of Bristol, Oakfield House, Oakfield Grove, Clifton, Bristol, BS8 2BN

**Keywords:** Cancer stem cells, cFLIP, TRAIL, Breast cancer

## Abstract

Therapeutic targeting of tumour initiating, cancer stem cells (CSCs), offers the potential to improve long-term responses to cancer treatments. The apoptosis related protein Cellular FLICE-Like Inhibitory Protein (cFLIP) has previously been reported to protect breast cancer cells and breast CSCs from the cytotoxic effects of chemotherapy and apoptosis-inducing agents.

We recently described the development of a small-molecule protein-protein inhibitor of cFLIP (OH14) that sensitizes refractory breast cancer cells to the death-receptor agonist Tumour Necrosis Factor Alpha Receptor Apoptosis Inducing Ligand (TRAIL).

Here we investigated whether the pharmacological inhibition of cFLIP also targeted breast CSCs.

Human breast cancer cell lines and primary-derived breast cancer samples were subjected to OH14 with or without TRAIL and assessed for bCSC viability by colony-formation and tumoursphere assay *in vitro* and xenograft tumour initiation *in vivo*. OH14 potentiated a reduction in the number of bCSCs after TRAIL treatment, mimicking the sensitizing effects previously observed with epigenetic silencing of cFLIP. Moreover, prolonged inhibition of cFLIP alone, either by shRNA knockdown or treatment with OH14, reduced the bCSC pool, an outcome that was independent of caspases.

These data provide proof-of-principle for the use of pharmacological inhibitors of cFLIP to target bCSCs and highlights for the first time both apoptosis-dependent and independent mechanisms for cFLIP-mediated regulation of the breast cancer stem cell pool.

## Introduction

Local tumour recurrence and metastasis arising after treatment remain significant challenges for improving long-term outcomes for cancer patients^1^. Both outcomes have been attributed to a minority sub-population of cancer cells with stem-like properties that are uniquely able to initiate tumour growth or re-growth^2^. Targeting of these so-called cancer stem cells (CSCs) has the potential to improve long-term responses in the management of cancer, yet treatment options are limited due to their inherent resistance to most therapeutic interventions, including immunotherapy^3,4^.

Tumour Necrosis Factor Alpha Receptor Apoptosis Inducing Ligand (TRAIL) is presented by immune cells to initiate death-receptor mediated apoptosis in target cells, as part of the body’s inherent tumour surveillance mechanism. TRAIL has previously been demonstrated to induce apoptosis preferentially in tumour cells^5^. Numerous clinical trials have attempted to adopt TRAIL agonists to treat different cancer types, however their efficacy has been limited by inherent resistance of the cancer cells to TRAIL^6,7^. This approach reflects an appreciation that future cancer treatments should consider tumour-microenvironment (TME) interactions alongside inherent tumour heterogeneity in their design, and that combined treatment modalities that sensitize malignant cells to TME surveillance mechanisms may ultiimately improve outcomes^8^.

One of a number of mechanisms of TRAIL resistance identified in cancer cells includes upregulation or stabilisation of the endogenous death-receptor associated protein Cellular FLICE-Like Inhibitory Protein (cFLIP), which also contributes to chemotherapy resistance in a variety of tumour types ^9–17^ and is itself a biomarker of poor prognosis^18^. There has been significant interest therefore in targeting cFLIP as a sensitizer of TRAIL or chemotherapeutic interventions^10,13,19^, yet until recently specific pharmacological targeting of cFLIP protein had proven difficult to achieve. However with a new-found molecular understanding of cFLIP’s mechanism of action ^10,20^, three different approaches to targeting cFLIP have come to light. These include the development of cFLIP interactors that stabilise caspase-8/cFLIPL heterodimers to promote caspase cleavage ^21,22^, and the development of small-molecule protein–protein inhibitors targeting either the DED2 domain of cFLIP^23^, or most recently its DED1 domain to disrupt FADD/caspase8 interactions^24^.

We and others have previously shown in breast cancer that inhibition of cFLIP sensitises cancer cells to TRAIL^19,25–27^ and moreover, preferentially eliminates breast CSCs (bCSCs)^27,28,12^, thus highlighting the TRAIL/cFLIP axis as an attractive therapeutic modality for targeting treatment-resistant intra-tumour heterogeity.

We recently reported on the sensitization of breast cancer cells to TRAIL cytotoxicity using a novel small-molecule cFLIP-DED1 inhibitor^24^, which we have termed OH14. Here we investigated the potential for OH14 to preferentially target bCSCs in the presence or absence or TRAIL, testing the hypothesis that pharmacological inhibition of cFLIP is an effective strategy to target CSCs in breast cancer.

## Materials and Methods

All experiments were performed with the approval of the Cardiff University School of Biosciences Ethics Committee (GM130-63, 2012 Dec 12). Animal experiments were performed under UK Home Office licence: PP1361991. Primary cells were derived from human breast tumour biopsies through the Wales Cancer Biobank^29^ which is funded by Health and Care Research Wales (SE Wales LREC approval 21_WA_0234) and from pleural effusions through the Manchester Cancer research Centre (MCRC) Biobank, UK under NRES Committee North West ethics approval 05/Q1402/25.

### Constructs

The pTRIPz cFLIP (cFLAR; RHS4696-201899832, Clone Id: V3THS_346945) and nonspecific control inducible shRNA lentiviral plasmids were purchased from Dharmacon/Horizon. The constitutive pLKO.1 sh_cFLIP and non-specific control vectors were kind gifts from Dr Ladislav Andera, Institute of Molecular Genetics, Prague^30^.

### Cell Lines

The human breast cancer cell lines MDA-MB-231^ER-HER2-^ HCC1954^HER2+^ and BT474 ^ER+HER2+^ were obtained from ATCC. MCF-7^ER+^ cell line was a gift from Dr Julia Gee, Cardiff University. SUM149 ^ER-HER2-^ cells were purchased from Asterand Bioscience (Detroit, USA). The primary-derived breast cancer pleural effusion cell lines, were a gift from Dr Rob Clarke, University of Manchester through the MCRC Biobank. The primary-derived cells generated from biopsy samples were obtained from the Wales Cancer Biobank^29^. All cell lines except SUM149 were cultured in RPMI 1640 medium (Invitrogen) supplemented with 10% foetal bovine serum (FBS) (Invitrogen), and 1% penicillin-streptomycin and L-glutamine mix (Invitrogen). The SUM 149 cell line was cultured in Hams F12 media (Invitrogen, Paisley, UK) supplemented with 5% fetal bovine serum (Sigma), 2mM L- glutamine (Invitrogen), 10mM HEPES (Invitrogen), 1μg/ml Hydrocortisone (Invitrogen) and 5μg/ml insulin (Invitrogen). All cell lines were cultured at 37°C in 5% CO_2_.

### Reagents

Recombinant soluble human TRAIL was purchased as super-killer TRAIL from Enzo Life Sciences. Unless otherwise stated, cells were treated with 20 ng/ml TRAIL without cross-linking for 18 hours. The pan-caspase inhibitor z-vad-fmk was purchased from R&D systems and used at a concentration of 20 μM. OH14 was used at the efficiacious and cFLIP-specific concentration of 100μM in all experiments, as previously determined by a combination of dose response studies and site-directed mutagenesis of the DED1 target domain of cFLIP^24^.

### Tumoursphere Formation Assay

Tumoursphere assays were carried out as described^27^ in non-adherent conditions in a serum-free epithelial growth medium (MEBM, Lonza), supplemented with B27 (Invitrogen), 20 ng/ml EGF (Sigma), 5 μg/ml Insulin (Sigma), and 25 μg/ml hydrocortisone (Sigma). Cells were plated in ultra-low attachment plates (Costar, Corning) at a density of 5000 cells/ml. After 7 days tumourspheres were counted, then collected by centrifugation (1100 rpm), dissociated in 0.05% trypsin, 0.25% EDTA (Invitrogen) and re-seeded at 5000 cells/ml for subsequent passages.

### Colony Forming Assay

Cells were seeded at a density of 185 cells/well in a 12-well plate format, so that cells were 50 per square cm, and cultured for 10 days before staining with Crystal violet/ethanol^28^. Colonies containing approximately 32 or more cells (having undergone 5 or more divisions) were counted using a GelCount platereader and software (Oxford Optronix).

### Flow Cytometry

Cells were trypsinised, washed and incubated for 1h in antibody diluted 1:100 in PBS. Flow cytometry was performed on an Accuri Flow Cytometer (BD Biosciences) and analysis of results was performed using a FlowJo software package. APC-conjugated CD44 antibody was purchased from BD Pharmingen, PE-conjugated ALDH1 antibody was purchased from Stratech. ALDH1 was also detected by the Aldefluor Assay according to the manufacturer’s instructions (StemCell Technologies).

### Tumour Initiation *in vivo*

Serial dilutions of untreated and OH14/TRAIL-treated cells were prepared in 50% Matrigel (BD Biosciences). Female athymic nude mice were randomly assigned to treatment groups and blinded to researchers assessing tumour development. The cell/Matrigel mix (75 μL) was injected above the lymph nodes of the fourth inguinal mammary fat pad (Envigo Life Sciences, UK). Mice were administered oestrogen ad-lib in their drinking water during the course of the experiment at a concentration of 10 μg/ml. Mice were culled when the entire control group developed tumours at least 5 mm in diameter.

### Western Blotting

Total cellular proteins were extracted from cultured cells and 30 μg analysed by Western blotting as described^28^. cFLIP antibodies used were purchased from Santa Cruz (5D8, sc136160) and Enzo Life Sciences (7F10, ALX-804-961-0100). To quantitate Western data, the pixel intensity of each band was quantified relative to its protein loading control (GAPDH, Santa Cruz, sc32233) by densitometry using ImageJ (http://imagej.nih.gov/ij/).

### Statistical Analysis

Throughout the article, data are represented as means with standard error from a minimum of three independent experiments, unless otherwise stated. Statistical significance was determined using a student’s T-test for two-paired samples. Key for statistical cut-offs on all graphs: * = p<0.05, ** = p<0.01, *** = p<0.001. L-Calc software was used to estimate stem cell number from serial dilutions of tumour xenografts: (http://www.stemcell.com/en/Products/All-Products/LCalc-Software.aspx).

## Results

### OH14 sensitizes breast cancer stem cells to TRAIL in vitro and *in vivo*

**Figure 1:**
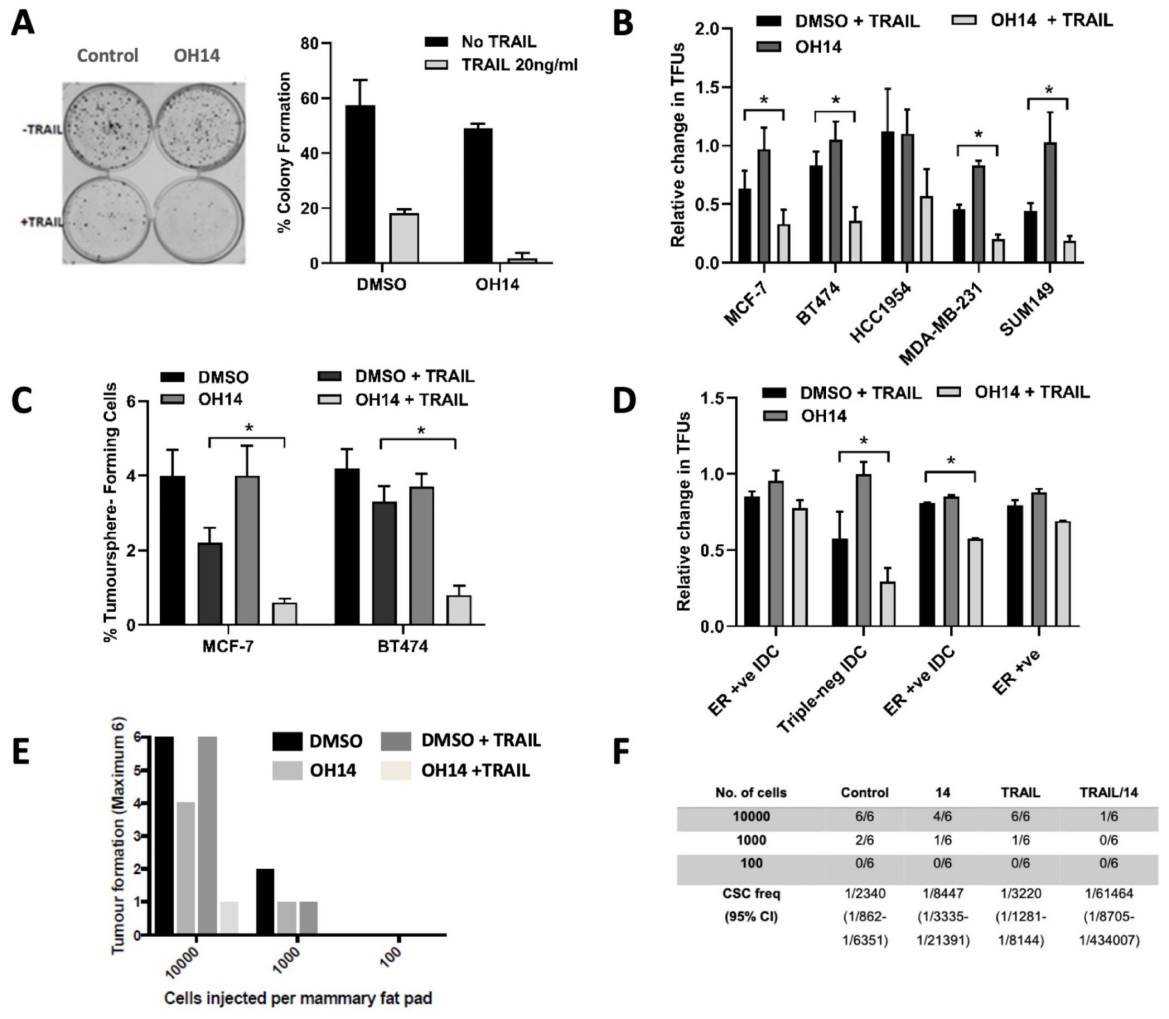
cFLIP inhibitor OH14 Sensitises breast cancer stem-like cells to TRAIL **A** MCF-7 cells were treated with 100μM OH14 followed by 20ng/ml TRAIL for 18 hours then assayed for colony formation. Error bars = standard error mean. **p<0.01 vs TRAIL alone. **B** A panel of breast cancer cell lines were treated with 100μM OH14 followed by 20ng/ml TRAIL for 18 hours then assayed for tumoursphere formation. Tumourspheres were counted 7 days later and data represented relative to DMSO control (TFU = tumoursphereforming unit) n=3. Error bars = standard error mean. *p<0.05 vs TRAIL alone, ***p<0.001 vs TRAIL alone; **C** MCF-7s and BT474s from B were passaged in the absence of OH14 and TRAIL and secondary tumourspheres quantified after a further 7 days. *p<0.05 vs TRAIL alone, **p<0.01 vs TRAIL alone. **D** A panel of primary pleural effusion-derived breast cancer cell lines were treated with 100μM OH14 followed by 20ng/ml TRAIL for 18 hours then assayed for tumoursphere formation. Tumourspheres were counted 7 days later, n=4 independent experiments. *p<0.05 vs TRAIL alone. **E** MDA-MB-231 were pre-treated with 100μM OH14 followed by 20 ng/ml TRAIL for 18 hours then harvested and implanted into the mammary fat pad of athymic nude mice at serial dilutions. The number of tumours formed relative to transplants was determined by palpation and confirmed by histological analysis at the end of the experiment. Tumour establishment was plotted for n=6 tumours per condition. **F**. Estimate of cancer stem cell numbers was calculated including 95% confidence limits.

We tested the effect of OH14 combined with TRAIL on the colony-forming (Figure 1a) and anoikis-resistant tumoursphere-forming (Figure 1b-d) cell subsets that are representative of breast cancer stem-like cells. OH14 significantly sensitized bCSCs to TRAIL-induced cytotoxicity in a selection of established and primary-derived cell lines representing different hormone receptor subtypes (Figure 1b-d). Combined OH14/TRAIL treatment was confirmed to target the self-renewing stem-progenitor population through serial passaging of pre-treated cells (Figure 1c). To support the clinical relevance of these findings, four primary cell lines derived from advanced breast cancers were treated with OH14/TRAIL for 18 hours then tested for tumoursphere-forming ability. OH14 significantly sensitised the tumoursphere-forming subpopulations to TRAIL in 2 out of 4 of these metastatic primary-derived breast cancer cell lines (Figure 1d). Notably, in all cell lines tested, short-term (18 hour) administration of OH14 alone had no effect on bCSC viability (Figure b-d). As the gold-standard assay for cancer stem cell properties is tumour formation in vivo, we assessed the ability of the OH14 and TRAIL combination to inhibit tumour initiation in a xenograft model. MDA-MB-231 cells were pre-treated with OH14 and TRAIL for 18 hours then viable cells sorted and transplanted orthotopically into the mammary fat pad of athymic nude mice at serial dilutions of 10^4^, 10^3^ and 10^2^ cells per transplant (Figure 1e). OH14 and TRAIL impaired tumour formation, depleting the tumour-initiating compartment 19-fold, from 1 in 3,220 following TRAIL treatment alone to 1 in 61,464 following combined OH14 and TRAIL treatment (Figure 1f).

### Long-term suppression of cFLIP alone reduces cancer stem cell viability

Taken together these data show that short-term chemical intervention of cFLIP DED1 interactions, sensitizes bCSCs to TRAIL mediated cytotoxicity, while in the absence of TRAIL, short-term use of OH14 had no effect on bCSC viability. These findings were consistent with our previous observations of transient (48h-72hr) epi-genetic silencing of cFLIP by siRNA^27^ and of the effects of OH14 in the ‘bulk’ (non-bCSC) cancer cell populatons^24^.

**Figure 2:**
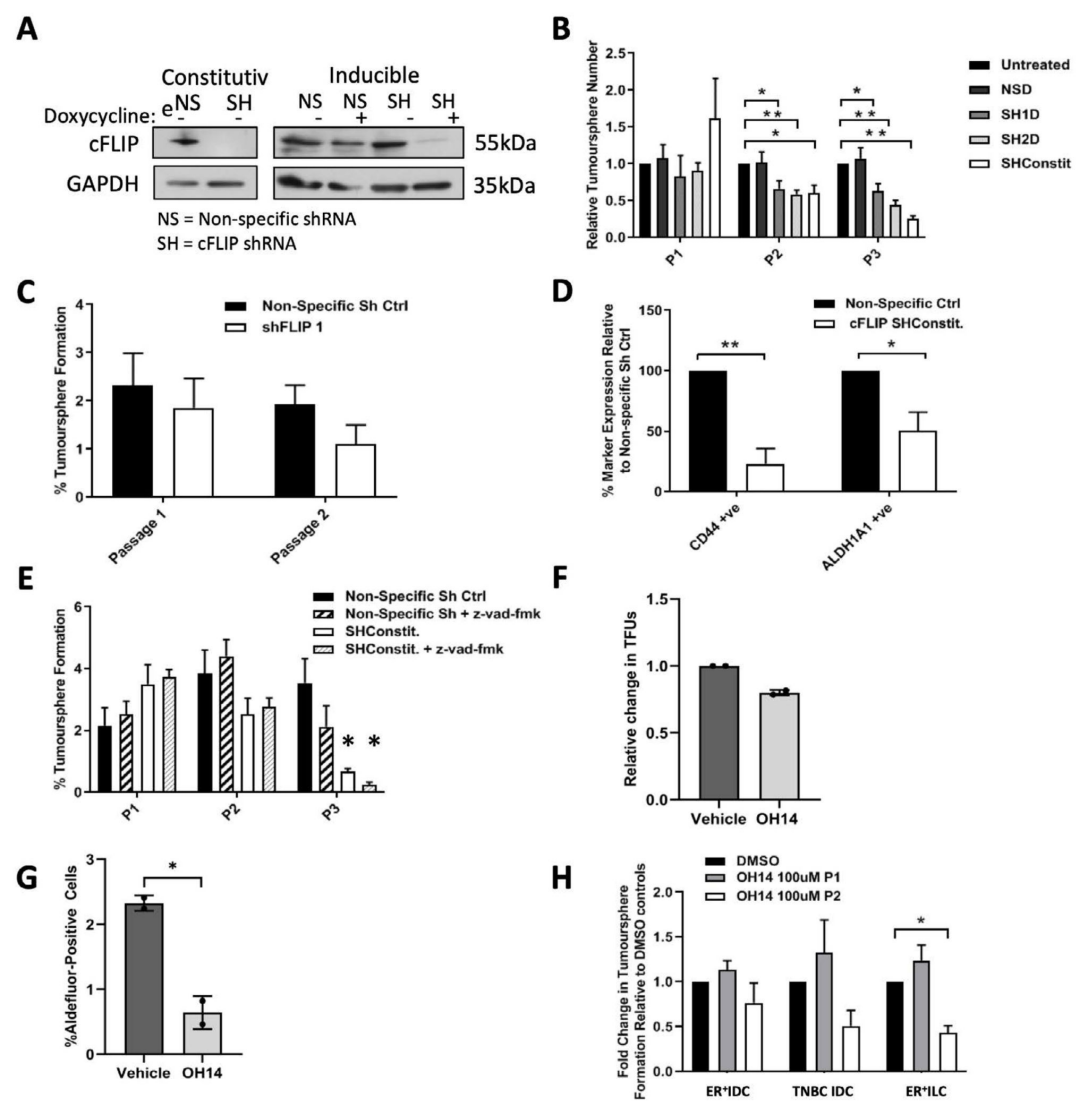
Long term inhibition of cFLIP alone reduces breast cancer stem-like cell viability A Generation of constitutive and inducible cFLIP shRNA cell lines reduces cFLIP levels: Inducible lines were treated with 10ug/ml doxycycline for 7 days and cFLIP levels determined by Western blotting. B MCF-7s expressing consititutive (SHconstit) or inducible (SH1 and SH2) shRNA targeting cFLIP were subjected to the tumoursphere assay: Inducible cell lines (SH1 and SH2) were treated with 10μg/ml doxycycline for 6 days prior to tumoursphere assay also in the presence of doxycycline. Tumourspheres were counted after 7 days and passaged. Data are compared to both untreated and non-specific (NS) shRNA controls (TFU = tumoursphere-forming unit) n=4 independent experiments *p<0.05 **p<0.01 C MDA-MB-231s expressing consititutive (SHconstit) shRNA targeting cFLIP were assayed for tumoursphere formation: Tumourspheres were counted after 7 days and passaged. Data are presented relative to non-specific (NS) shRNA control (TFU = tumoursphere-forming unit) n=3 independent experiments D MCF-7s expressing consititutive (SHconstit) shRNA targeting cFLIP vs. a non-specific (NS) shRNA control were assayed for expression of stem cell markers CD44 or ALDH1A by flow-cytometry. n=3 independent experiments *p<0.05 **p<0.01 E MCF-7s expressing consititutive (SHconstit) shRNA targeting cFLIP vs. a non-specific (NS) shRNA control were assayed for tumoursphere formation in the presence or absence of 20μM pan-caspase inhibitor z-vad-fmk. Tumourspheres were counted after 7 days and passaged. (TFU = tumoursphere-forming unit) n=4 independent experiments *p<0.05 versus respective non-specific controls F MCF-7 cells were treated with 100μM OH14 for 6-days prior to tumoursphere assay. Tumourspheres were counted after 7 days. Average of two independent experiments shown, datapoints = individual repeats, error bars = standard deviation of 2 biological repeats G MCF-7 cells were treated with 100μM OH14 for 6-days then expression of the stem cell marker ALDH1A was determined by flow-cytometry (Aldefluor Assay). Average of two independent experiments shown, error bars = standard deviation of 2 biological repeats. H Primary biopsy breast cancer cells were treated with 100μM OH14 for 7-days prior to tumoursphere assay also in the presence of OH14. Tumourspheres were counted after 7 days and passaged n=3 independent experiments *p<0.05.

However, we and others have shown previously that inhibition of cFLIP impairs Wnt signalling^28,31,32^. As Wnt/beta-catenin is a key pathway in promoting CSC self-renewal we wanted to test the hypothesis that prolonged inhibition of cFLIP could impact on the long-term self-renewal capacity and thus viability of bCSCs. To do this we generated MCF-7 and MDA-MB-231 cell lines expressing inducible or consititutive shRNA vectors targeting cFLIP (Figure 2a). Sustained inhibition of cFLIP by shRNA significantly impaired tumoursphere formation across passages in both MCF-7s (Figure 2b) and MDA-MB-231 (Figure 2c). In addition stable shRNA cFLIP also reduced the number of cells expressing the stem cell-associated markers CD44 and ALDH (Figure 2d). Importantly, loss of tumoursphere-forming ability by cFLIP shRNA could not be rescued by treatment with the pan-caspase inhibitor z-vad-fmk, confirming our previous conclusion that this was not due to induction of apoptosis (Figure 2E). Similar to shRNA inhibition, treatment of MCF-7 cells for 6 days with OH14 resulted in a reduction in tumoursphere formation (Figure 2f) and ALDH marker expression (Figure 2g). Furthermore, prolonged treatment with OH14 reduced tumoursphere formation in primary-derived breast cancer cells (Figure 2h). Taken together these data suggest that sustained inhibition of cFLIP, either epigenetically or by OH14, impairs CSC viability independently of its role in extrinsic apoptosis signalling.

## Discussion

We have previously shown that inhibition of cFLIP by siRNA sensitises breast CSCs to TRAIL to a much greater extent than the non-stem population^27^. We now demonstrate that breast CSCs can also be targeted pharmacologically with the use of a small molecule protein:protein interaction inhibitor of cFLIP, OH14. OH14 was developed as an inhibitor of cFLIP binding to the caspase8/FADD complex formed upon TRAIL activation, and this function has been confirmed experimentally^24,33^. It is predicted that other pharmacological inhibitors of cFLIP with alternate modes of action would exhibit the same outcomes on bCSC activity^21,23^. OH14 sensitised CSCs to TRAIL in each cell line tested, even in those that had previously been shown to be refractory to TRAIL (eg. BT474 and SUM149)^28^. In addition to the sensitizing effects, we also report here that long-term suppression of cFLIP, either through epigenetic repression of expression, or pharmacological intervention of protein binding, leads to a reduction in ‘stemness’ characteristics in the cancer cell population *in vitro* in the absence of TRAIL. This effect was independent of caspases suggesting that an alternative mechanism, possibly involving self-renewal may be responsible. Our previous work showing the involvement of cFLIP in the Wnt signalling pathway is one potential mechanism by which this could occur^28^ as disruption of Wnt signalling has been shown to be detrimental to breast CSC self-renewal and controls breast cancer aggressiveness^34,35^.

At present, these data demonstrate proof of principle for the pharmacological targeting of cancer stem cells via cFLIP. The majority of anti-cancer agents currently in development that have been shown to target CSCs are inhibitors of cell-surface receptors such as EpCAM (e.g. adecatumumab) or CSC-related pathways including Wnt signalling^4,36^. Here we show that pharmacological inhibition of a single target, cFLIP, can impair CSCs by more than one mechanism (that is, via apoptosis-dependent and independent pathways), thus increasing its potential for efficacy.

While the key finding of this study is the successful pharmacological inhibition of cancer stem cell activity via selective targeting of cFLIP, a limitation of this particular inhibitor is that this was only achievable at relatively high micromolar concentrations. Therefore, we do not anticipate that OH14 in its current form will be a viable drug for clinical use but will instead provide a useful pre-clinical tool and a platform for further development, with the hope of identifying a clinically viable structural analogue. In this study we have further considered the clinical relevance of a cFLIP inhibitor by testing OH14 on a panel of breast cancer cell lines and primary derived tumours representative of different tumour subtypes. Although the OH14/TRAIL combination was able to target each of the established and primary cell line tested, the degree of TRAIL sensitisation varied, with the greatest responses observed in the triple negative breast cancer cells (Figure 1B,D). Despite this there was no clear statistical association between OH14-response and breast tumour subtype, given the small number of primary samples studies here. We have previously demonstrated the importance of cFLIP in determining tumour sensitivity to TRAIL using cell line and primary derived xenograft models of endocrine resistant breast cancer^12^. A more extensive study of primary samples from breast tumours and also other cancer types, including primary derived xenografts, would allow for a greater understanding of cFLIP-dependent subtype specificity and clinical relevance.

## References

[1] Sonkin D, Thomas A, Teicher BA (2024). Cancer treatments: Past, present, and future. Cancer Genet.

[2] Meacham CE, Morrison SJ (2013). Tumour heterogeneity and cancer cell plasticity. Nature.

[3] Agudo J, Miao Y (2025). Stemness in solid malignancies: coping with immune attack. Nat Rev Cancer.

[4] Du FY, Zhou QF, Sun WJ (2019). Targeting cancer stem cells in drug discovery: Current state and future perspectives. World J Stem Cells.

[5] Walczak H, Miller RE, Ariail K (1999). Tumoricidal activity of tumor necrosis factor-related apoptosis-inducing ligand in vivo. Nat Med.

[6] Deng D, Shah K (2020). TRAIL of Hope Meeting Resistance in Cancer. Trends Cancer.

[7] Maji A, Paul A, Sarkar A (2024). Significance of TRAIL/Apo-2 ligand and its death receptors in apoptosis and necroptosis signalling: Implications for cancer-targeted therapeutics. Biochem Pharmacol.

[8] Liu H, Dilger JP (2025). Different strategies for cancer treatment: Targeting cancer cells or their neighbors?. Chin J Cancer Res.

[9] Haghir-Sharif-Zamini Y, Khosravi A, Hassan M (2025). c-FLIP/Ku70 complex; A potential molecular target for apoptosis induction in hepatocellular carcinoma. Arch Biochem Biophys.

[10] Ivanisenko NV, Seyrek K, Hillert-Richter LK (2022). Regulation of extrinsic apoptotic signaling by c-FLIP: towards targeting cancer networks. Trends Cancer.

[11] Kim JH, Lee J, Im SS (2024). Glutamine-mediated epigenetic regulation of cFLIP underlies resistance to TRAIL in pancreatic cancer. Exp Mol Med.

[12] Piggott L, Silva A, Robinson T (2018). Acquired Resistance of ER-Positive Breast Cancer to Endocrine Treatment Confers an Adaptive Sensitivity to TRAIL through Posttranslational Downregulation of c-FLIP. Clin Cancer Res.

[13] Safa AR (2022). Drug and apoptosis resistance in cancer stem cells: a puzzle with many pieces. Cancer Drug Resist.

[14] Wang WD, Shang Y, Wang C (2022). c-FLIP promotes drug resistance in non-small-cell lung cancer cells via upregulating FoxM1 expression. Acta Pharmacol Sin.

[15] Wei L, Kim SH, Armaly AM (2024). HuR inhibition overcomes cFLIP-mediated doxorubicin resistance in triple-negative breast cancer. NPJ Precis Oncol.

[16] Shahriari Felordi M, Alikhani M, Farzaneh Z (2023). (-)-Epigallocatechin-3-gallate induced apoptosis by dissociation of c-FLIP/Ku70 complex in gastric cancer cells. J Cell Mol Med.

[17] Yerbes R, Lopez-Rivas A, Reginato MJ (2012). Control of FLIP(L) expression and TRAIL resistance by the extracellular signal-regulated kinase1/2 pathway in breast epithelial cells. Cell Death Differ.

[18] Zang F, Wei X, Sun B (2014). [Relationship of c-FLIP(L) protein expression with molecular subtyping and clinical prognosis in invasive breast cancer]. Zhonghua Bing Li Xue Za Zhi.

[19] Yerbes R, Lopez-Rivas A (2012). Itch/AIP4-independent proteasomal degradation of cFLIP induced by the histone deacetylase inhibitor SAHA sensitizes breast tumour cells to TRAIL. Invest New Drugs.

[20] Majkut J, Sgobba M, Holohan C (2014). Differential affinity of FLIP and procaspase 8 for FADD’s DED binding surfaces regulates DISC assembly. Nat Commun.

[21] Hillert LK, Ivanisenko NV, Busse D (2020). Dissecting DISC regulation via pharmacological targeting of caspase-8/c-FLIP(L) heterodimer. Cell Death Differ.

[22] Hillert-Richter LK, Konig C, Ivanisenko NV (2024). Targeting caspase-8/c-FLIP(L) heterodimer in complex II promotes DL-mediated cell death. Front Cell Dev Biol.

[23] Yaacoub K, Pedeux R, Lafite P (2024). The Identification of New c-FLIP Inhibitors for Restoring Apoptosis in TRAIL-Resistant Cancer Cells. Curr Issues Mol Biol.

[24] Giancotti G, French R, Hayward O (2025). The Discovery of Small-Molecule Inhibitors of CFLIP That Sensitise Tumour Cells to TNF-Related Apoptosis-Inducing Ligand. Academia Oncology.

[25] Frew AJ, Lindemann RK, Martin BP (2008). Combination therapy of established cancer using a histone deacetylase inhibitor and a TRAIL receptor agonist. Proc Natl Acad Sci U S A.

[26] Holmgren C, Thornberg Sunstrom E, Granqvist V (2022). Induction of Breast Cancer Cell Apoptosis by TRAIL and Smac Mimetics: Involvement of RIP1 and cFLIP. Curr Issues Mol Biol.

[27] Piggott L, Omidvar N, Perez Marti S (2011). Suppression of apoptosis inhibitor c-FLIP selectively eliminates breast cancer stem cell activity in response to the anti-cancer agent, TRAIL. Breast Cancer Res.

[28] French R, Hayward O, Jones S (2015). Cytoplasmic levels of cFLIP determine a broad susceptibility of breast cancer stem/progenitor-like cells to TRAIL. Mol Cancer.

[29] Parry-Jones ASL (2018). The Wales Cancer Bank (WCB). Open Journal of Bioresources.

[30] Vinarsky V, Krivanek J, Rankel L (2013). Human embryonic and induced pluripotent stem cells express TRAIL receptors and can be sensitized to TRAIL-induced apoptosis. Stem Cells Dev.

[31] Katayama R, Ishioka T, Takada S (2010). Modulation of Wnt signaling by the nuclear localization of cellular FLIP-L. J Cell Sci.

[32] Naito M, Katayama R, Ishioka T (2004). Cellular FLIP inhibits beta-catenin ubiquitylation and enhances Wnt signaling. Mol Cell Biol.

[33] Hughes MA, Powley IR, Jukes-Jones R (2016). Co-operative and Hierarchical Binding of c-FLIP and Caspase-8: A Unified Model Defines How c-FLIP Isoforms Differentially Control Cell Fate. Mol Cell.

[34] Castagnoli L, Tagliabue E, Pupa SM (2020). Inhibition of the Wnt Signalling Pathway: An Avenue to Control Breast Cancer Aggressiveness. Int J Mol Sci.

[35] Katoh M (2017). Canonical and non-canonical WNT signaling in cancer stem cells and their niches: Cellular heterogeneity, omics reprogramming, targeted therapy and tumor plasticity (Review). Int J Oncol.

[36] Ervin EH, French R, Chang CH (2022). Inside the stemness engine: Mechanistic links between deregulated transcription factors and stemness in cancer. Semin Cancer Biol.

